# Streamline-Based Analysis: A novel framework for tractogram-driven streamline-wise statistical inference

**DOI:** 10.21203/rs.3.rs-10123464/v1

**Published:** 2026-07-06

**Authors:** Simone Zanoni, Jinglei Lv, Sharon L. Naismith, Robert E. Smith, Fernando Calamante

**Affiliations:** 1. School of Biomedical Engineering, The University of Sydney, Sydney, Australia; 2. Brain and Mind Centre, The University of Sydney, Sydney, Australia; 3. Sydney Medical School, The University of Sydney, Sydney, Australia; 4. School of Psychology, The University of Sydney, Sydney, Australia; 5. The Florey Institute of Neuroscience & Mental Health, Melbourne, Australia; 6. Florey Department of Neuroscience and Mental Health, The University of Melbourne, Melbourne, Australia; 7. Sydney Imaging, The University of Sydney, Sydney, Australia

## Abstract

Most diffusion MRI studies of white matter are interpreted in terms of anatomically defined fibre bundles, yet current statistical frameworks fail to simultaneously provide high-resolution pathway-level localisation, whole-brain coverage, and good statistical power with family-wise error control. We introduce Streamline-Based Analysis (SBA), a novel framework that performs statistical inference directly on individual tractography streamlines. SBA flexibly accommodates any imaging-derived, streamline-wise quantitative metric leveraging streamline similarity to perform streamline-wise data smoothing and statistical enhancement within a non-parametric permutation testing framework that controls family-wise error. Applied to healthy ageing, SBA recapitulated established patterns observed with Fixel-Based Analysis and uncovered previously unreported effects, while yielding more spatially coherent, pathway-level effects with improved anatomical interpretability. By achieving pathway-level specificity, whole-brain coverage, and family-wise error control, SBA fills a key analytical gap between voxel-, fixel-, and tract-level methods, providing a flexible framework for detecting white matter effects across diverse diffusion MRI studies.

## Introduction

The human brain comprises a complex network of White Matter (WM) fibre bundles that support sensory, cognitive, motor, and other functions. Diffusion Magnetic Resonance Imaging (dMRI) provides a powerful tool for non-invasive characterisation of WM structures in vivo by enabling the modelling of microstructural properties and the reconstruction of WM pathways^1^. dMRI analyses capture WM organisation across complementary spatial scales, ranging from individual voxels and fixels (defined as individual fibre populations within a voxel)^2^ to long-range tracts that connect distant grey matter regions. As a result, dMRI has become a central tool in neuroscience, neurology, and psychiatry. By enabling systematic characterisation of WM structure and its variability across individuals and populations, dMRI facilitates the study of how brain connectivity develops^3,4^, changes with ageing^5,6^, and is disrupted by diseases^7–10^.

Current statistical approaches for analysing dMRI data operate at distinct spatial scales. Among commonly used frameworks, voxel-based methods assess microstructural metrics at each WM voxel^11^, with Tract-Based Spatial Statistics (TBSS) restricting the analysis to a core WM skeleton^12^. Fixel-Based Analysis (FBA) provides greater specificity by attributing quantitative measures and statistical significance to resolved individual fibre populations within WM voxels^13^. At a coarser level, segment-wise approaches aggregate voxel- or fixel-derived measures within predefined regions or bundles, yielding bundle-level statistics^14,15^.

These methods reflect a trade-off between spatial specificity, statistical sensitivity, and anatomical scope. Voxel- and fixel-wise analyses maximally preserve local detail but incur a substantial multiple-comparison burden. To retain inferential power, these methods rely on statistical enhancement strategies^2,16^ and employ permutation-based non-parametric inference^17^ to control family-wise error rate. In contrast, TBSS and tract-level methods improve statistical sensitivity by reducing dimensionality, at the cost of narrowing spatial coverage or sacrificing granularity of localisation^18^.

Of note, research questions addressed with dMRI are usually related to anatomically defined fibre bundles, which provide the interpretative landscape through which neuroscientific and clinical findings are typically framed. However, existing methods do not simultaneously offer: (a) high-resolution localisation of pathways; (b) whole-brain coverage with bundle-related interpretability; and (c) sufficient statistical sensitivity to detect structured, pathway-level effects.

To address this unmet need, we propose *Streamline-Based Analysis* (SBA), a statistical inference framework that enables streamline-wise specificity across a whole-brain tractogram, providing spatially precise, statistically robust, and anatomically interpretable characterisation of WM changes. We demonstrate its utility in healthy ageing, where SBA uncovers previously unreported, spatially coherent pathway-related changes alongside established WM ageing patterns. We further highlight its broader potential for investigating pathway-resolved WM alterations across diverse neuroscientific and clinical applications.

## Results

**Fig 1.**
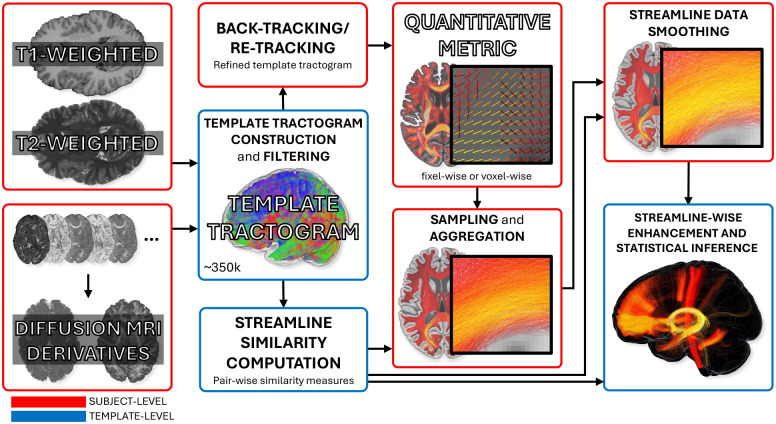
Overview of the SBA pipeline. Multimodal MRI data are used to construct a study-specific tissue-unbiased template tractogram (which is first filtered and subsequently refined for each subject, while retaining streamline-to-streamline correspondence); this defines a tractogram-based coordinate system for streamline-wise analysis. Imaging measures are sampled and aggregated into streamline-wise metrics. Streamline similarity is used to enable both streamline-wise data smoothing and statistical enhancement within a permutation-based inference framework.

SBA achieves statistical effects at the level of individual streamlines within a tractogram through a structured pipeline (Fig. 1), where a template tractogram represents the coordinate system within which quantification and statistical analysis is performed to ensure inter-subject correspondence for each streamline. Subject-specific imaging measures can be flexibly sampled and aggregated into streamline-wise metrics, allowing the choice of the quantitative measure and summary statistic to be tailored to the specific research question. SBA involves the definition of streamline similarity, which echoes the notion of voxel adjacency in Voxel-Based Analysis (VBA) and streamline-based connectivity in FBA^2^; this is exploited for both data smoothing and tailored statistical enhancement to improve sensitivity to coherent effects within a non-parametric framework. SBA enables pathway-specific localisation, high-resolution inference, and whole-brain coverage, within a single analysis framework.

### Template tractogram as a coordinate system for statistical analysis

Following standard image preprocessing, SBA establishes a common space across the study cohort through study-specific tissue-unbiased template tractogram construction^19^. Spherical-deconvolution Informed Filtering of Tractograms (SIFT)^20^ was applied in template space to reduce redundancy in the initial 1M-streamline set, yielding a template tractogram containing approximately 350k streamlines providing homogeneous coverage of WM trajectories.

**Fig. 2.**
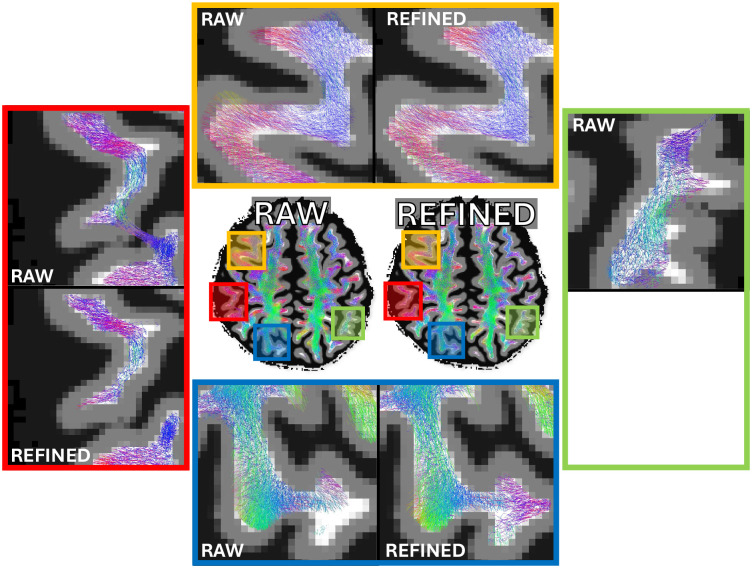
Trajectory refinement improves streamline/local anatomy alignment in subject space. Qualitative comparison between template streamlines projected to subject space (“raw”) and their “refined” back-tracking/re-tracking version in four magnified regions from a slice of a representative subject. Streamlines are overlaid on tissue segmentation to provide high anatomical contrast (Cerebro Spinal Fluid – CSF; cortical Gray Matter – cGM; White Matter - WM). The refinement effect of this procedure can be observed in the improvements of regions with lack of WM coverage, and elimination of spurious cGM and CSF incursions.

Once transformed from template to subject space, streamline trajectories were refined to better align to subject-specific anatomy and improve sampling of image data, using a new back-tracking/re-tracking procedure (see Methods for details). This approach allows distal streamline trajectories to adapt to local anatomy and better represent local fibre geometry within each subject, while preserving one-to-one streamline correspondence across subjects (see Supplementary Information for implementation details). Qualitative assessment shows that this refinement substantially improves the conformity of template streamlines to subject-specific anatomy and its tissue segmentation^21,22^ (Fig. 2). Although tractograms generated in subject space (“native”) are not used within the SBA pipeline, they provide a useful benchmark for evaluating tractogram morphology in subject space. To quantify the performance of the refinement procedure, we used the Track Density Imaging (TDI) approach^23^, which enables mapping the local streamline density onto a 3D grid. Across the cohort, the refined tractograms exhibit a marked reduction in Mean Square Error relative to the native ones compared to the template tractograms naively projected to subject space (mean ± standard deviation=10.90 ± 0.87 vs 17.58 ± 2.66).

### Streamline-wise quantitative metrics

A central component of SBA is the flexible definition of the streamline-wise metric quantified, upon which inference is performed. For each subject, an imaging measure is mapped onto refined streamlines in subject space and aggregated into per-streamline quantities. Similarly to the Track-Weighted Imaging framework^24^, this allows using any quantitative metric and aggregation strategy appropriate for the analysis. In this work, we demonstrate SBA using a streamline-wise analogue of the Fibre Density and Cross-section (FDC) metric from the FBA framework^25^, which we refer to as “sFDC”. This is obtained by averaging the fixel-wise Fibre Density (FD) (a measure of intra-axonal volume) and Fibre Cross-section (FC) (which captures morphological properties) information along the trajectory of each streamline and combining them into a single biologically interpretable metric that is sensitive to both microstructural and macrostructural characteristics of WM fibre bundles.

### Streamline similarity

**Fig. 3.**
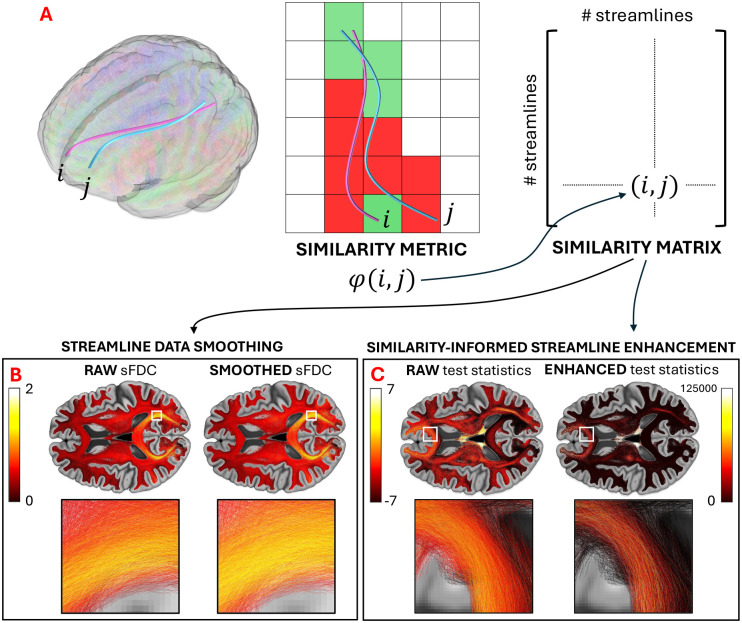
Conceptual overview of the streamline similarity computation and its applications. **A**: illustration of spatial overlap of streamlines i and j, where green denotes shared voxel grid elements and red exclusive ones, and consequent population of the streamline similarity matrix. **B**: representative case of similarity-informed smoothing of sFDC streamline-wise data. **C**: Depiction of the effect of Similarity-informed Streamline Enhancement (SSE) on a test statistic tractogram (note different test-statistic colourmap scales in C between raw and enhanced tractogram).

In VBA and FBA, voxel adjacency and streamlines-based fixel-fixel connectivity respectively are used to define “proximity” between image elements for subsequent calculations. To achieve the same for quantitative data defined on streamlines, SBA utilises a pair-wise similarity metric based on the shared spatial extent of streamline trajectories. The measure is conceptually similar to previous overlap-based metrics in the context of tractogram clustering^26–28^: each streamline is encoded according to the voxels it traverses (Fig. 3A), allowing to compute the proportions of streamline trajectories that overlap in 3D space. This yields a measure of similarity between all pairs of streamlines, reflecting both shared trajectory and spatial proximity (see Supplementary Video 1 for the relationship between cohort of similar streamlines and similarity metric).

### Streamline data smoothing

Smoothing of subject data prior to statistical inference is typically performed in neuroimaging to improve data normality and encourage formation of supra-threshold clusters of elements. In a manner conceptually similar to Gaussian smoothing in VBA, where the weighted mean computed per voxel applies greater weight to nearby voxels and progressively lesser weights to more distant ones, we adopt an analogous strategy in SBA, where smoothing weights are governed by similarity values between streamlines. Smoothing reduces local variations in streamline-wise measurements (e.g. sFDC) (Fig. 3B).

### Similarity-informed streamline enhancement

We introduce Similarity-informed Streamline Enhancement (SSE), a statistical enhancement procedure derived from Threshold-Free Cluster Enhancement (TFCE)^16^. SSE takes streamline-wise test-statistics and boosts the belief in the presence of statistical effects associated with streamlines based on the support provided by proximal streamlines. Specifically, the enhancement depends on both the magnitude of the effect in that streamline and the cardinality of proximal streamlines similarly reporting statistical effect (see Methods for more details).

### Statistical inference

SBA employs SSE within an established non-parametric permutation testing framework^17^ to obtain streamline-wise p-values with Family-Wise Error (FWE) control. In this procedure, data are suitably shuffled under the corresponding null hypothesis to compute a test statistic per streamline, to which SSE is then applied; the set of maximal enhanced test statistics across many such shuffles form a non-parametric null distribution against which the empirical data are compared to establish statistical significance.

### Example application

**Fig. 4.**
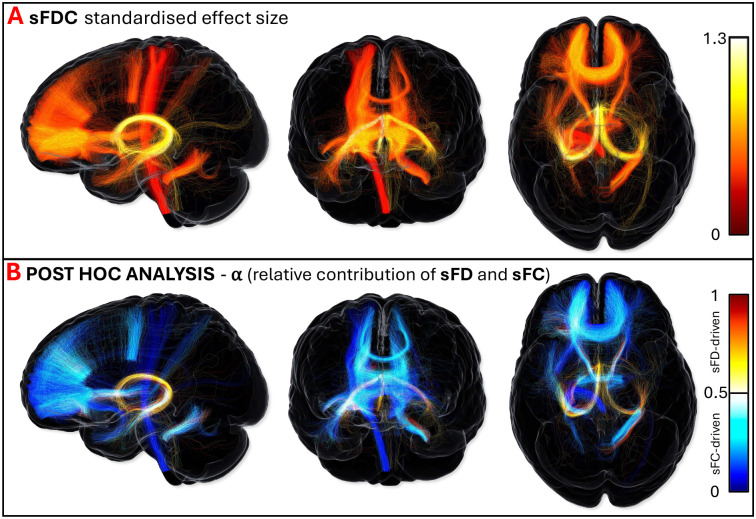
Outcomes of the SBA of sFDC and post-hoc analysis for the comparison between mature and old groups. **A**: streamlines with significant (p-value < 0.05, sample size n=60) differences, colour-coded by their standardised effect size. **B**: The same set of significant streamlines colour-coded by parameter α, indicating the relative influence of along-streamline average FD (sFD) and FC (sFC) to the sFDC effect. Left to right: sagittal, coronal and axial projections.

We demonstrate the utility of the SBA framework by assessing healthy ageing-related differences in streamline-wise sFDC when comparing “mature” (n=30, 35–40 years) and “old” (n=30, 70–75 years) groups, using data from the Human Connectome Project in Aging (HCP-A)^29^. Streamlines within several major WM bundles, including projection, association and commissural tracts, showed significantly reduced sFDC in the older group (Fig. 4A; see also Supplementary Video 2). Internal SBA parameters for this analysis were selected through a series of simulation experiments (reported in full in the Supplementary Information).

For the composite sFDC metric, the contribution of constituent measures (i.e. along-streamline averaged FD and FC) were disentangled post-hoc, providing a measure of their relative influence without additional statistical testing^30^. To do this, we compared the computed group differences for each component and determined their relative contribution, yielding an index denoted as α, where 0.0 indicates an effect in sFDC driven exclusively by an effect in FC, and 1.0 indicates an sFDC effect driven exclusively by FD. Figure 4B shows SBA significant streamlines colour-coded by α: most detected bundles have an FC dominated effect (i.e. alpha<0.5), with some exceptions (e.g. bilateral fornix), which had an effect primarily driven by FD (Supplementary Video 2).

**Fig. 5.**
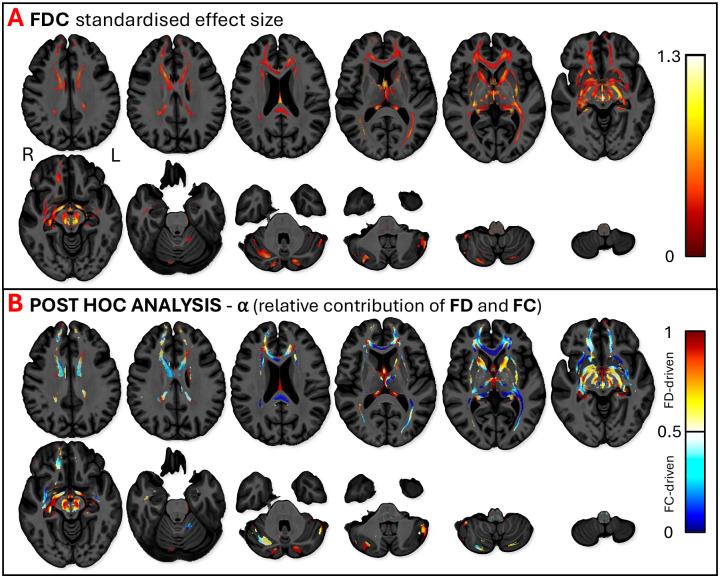
Outcome of the Fixel-Based Analysis of FDC and post hoc analysis results for the comparison between mature and old groups. Results are overlaid onto T1-weighted axial slices evenly spaced from superior to inferior. **A**: fixels with significant differences (p-value<0.05, sample size n=60) are colour-coded by standardised effect size. **B**: significant fixels colour-coded by parameter α, indicating the relative influence of FD and FC to the FDC effect.

For visual comparison, we also present the results from the widely used FBA of FDC^25^ of the same data. Figure 5A illustrates fixels that showed significant decrease in FDC in the old group relative to the mature group colour-coded by standardised effect size. Figure 5B shows their α values, indicating the relative contribution of FD and FC to the observed change in FDC^30^. The interpretation of α is analogous for FBA and SBA: in FBA the metrics are per-fixel, whereas in SBA the components correspond to along-streamline averaged quantities.

The spatial patterns detected by SBA broadly matched those obtained with FBA applied to FDC, reflecting the fact that SBA evaluates a streamline-aggregated representation of the same underlying fixel-wise metric. While both frameworks capture broadly concordant age-related alterations in combined WM micro- and macrostructural properties, observed differences may arise from the distinct spatial representations and statistical enhancement underlying the two methods. Both SBA and FBA revealed maximal standardised effects within the fornix bilaterally; this finding is consistent with well-established ageing-related vulnerability of hippocampal–diencephalic pathways^31–33^. Several major commissural and projection pathways showed significant effect in both methods, including the anterior corona radiata, forceps minor, superior frontal fasciculus, rostral corpus callosum, inferior fronto-occipital fasciculus (right), corona radiata superior (left), and the stria terminalis; these regions represent the core overlap between SBA and FBA and are broadly concordant with previous voxel-based and fixel-level findings^31,32^. Note, however, that while SBA natively attributes these outcomes to complete pathways, FBA yields clusters of significant fixels that typically only partially cover the full extent of the tracts, necessitating subsequent subjective assignment to those bundles.

Findings specifically attributable to SBA were in the right corticospinal tract, which has not been reported before using other methods, and more spatially extended effects than FBA within the anterior corona radiata and the genu of the corpus callosum. This may indicate increased sensitivity to the presence of pathway-level changes in those regions, in contrast to FBA which exhibited more fragmented effects, making it difficult to confidently associate FBA findings with a single tract. Conversely, FBA showed greater anterior spread within the body of the corpus callosum, and a visibly more extended cluster in the optic tract and the anterior commissure; here SBA yielded more restricted, yet still interpretable, effects.

The post-hoc decomposition of FD and FC contributions produced qualitatively different interpretations for SBA and FBA, with FBA-derived indices not entirely coherent at the macroscale within tracts (e.g. genu of the corpus callosum). This increased instability was most evident near the tissue boundaries, where FC is influenced by higher-frequency non-linear deformations and FD is influenced by residual image misalignment and differences in cortical folding. While ageing-related reductions in sFDC predominantly arose from a reduction in FC, concordant with the known brain atrophy associated with ageing, SBA revealed a localised and clear FD-driven effect in the fornix; this might be the result of ventricular enlargement causing increasing density in those areas^34^. For FBA the FD predominant relative contribution extended beyond the periforniceal region into the broader mesencephalic region. Conversely, FC driving effect was more evident in the frontal lobe, as well as in association and projection fibres.

## Discussion

We introduce Streamline-Based Analysis (SBA), a novel framework that performs whole-brain statistical inference directly on individual streamlines as the fundamental analysis unit (cf. existing voxel-wise, fixel-wise, and bundle-wise approaches). We demonstrate how SBA enables pathway-specific localisation, facilitating a more direct attribution of effects to specific WM bundles. In the example application of SBA illustrated here to characterise ageing-related WM differences, we demonstrate that streamline-wise inference provides spatially coherent, interpretable, and statistically robust results, while maintaining full coverage of the brain WM. Although comparison with other frameworks can offer useful context, and FBA has been included here as a widely used and related framework, the key contribution of this work lies in proposing SBA as a self-contained analytical approach with distinct conceptual and methodological advantages, which can complement existing analysis methods.

Given the large number of statistical tests inherent to whole-brain inference, SBA required purposely designed strategies for smoothing and statistical enhancement, analogous to those required in voxel-based^16^ and fixel-based analyses^2^. To this end, we defined a streamline similarity metric that characterises the neighbourhood of a streamline. The voxel-intersection-based similarity metric used here was selected for its efficiency and interpretability: it captures shared spatial occupancy without requiring computationally heavy operations on the full set of streamline vertices, which can be prohibitive if necessitating comparison of all streamline pairs. This metric is however a substitutable modular component of SBA. This similarity measure enables firstly smoothing of streamline-associated measurements with the values associated with nearby streamlines proportional to the similarity of their trajectories, and secondly a novel dedicated statistical enhancement procedure (SSE) which is embedded within a non-parametric permutation testing framework that provides statistical power with rigorous FWE rate control^17^.

Sharing a common underlying principle with Track-Weighted Imaging^24^, SBA offers substantial flexibility in how streamlines can be used to sample diverse imaging features for subsequent statistical inference. While the ageing application shown here used the composite metric sFDC as an exemplar, SBA is compatible with a wide range of streamline-aggregated metrics, either derived from MRI or other (co-registered) modalities. Focusing on the endpoints of the streamline also offers a natural way to combine WM and GM information in a joint analysis, such as for multi-modal structural-functional analysis (cf. track-weighted functional connectivity methods^35,36^). The expression by which quantitative values sampled at each streamline vertex are aggregated to a single scalar value per streamline (e.g. in the ageing example shown here, the average value) is also under experimental control.

A practical consideration in SBA is the size and density of the tractogram. While ~350k streamlines may appear modest in comparison with other tractography studies (e.g. connectome studies often utilise millions of streamlines per participant), factors contributing to the choice of this number are somewhat different for SBA than other contexts. For example, in connectome-based analyses, increasing the number of streamlines improves the robustness of edge-wise connectivity estimates by increasing the cardinality of the aggregation. In SBA, each streamline contributes a *unique* unit of quantification and statistical testing; beyond a certain density, additional streamlines largely follow highly similar trajectories within the same bundles and therefore contribute little new information when sampling from the same underlying image, while substantially increasing the dimensionality of the multiple-comparison problem. Furthermore, the streamline-streamline similarity matrix grows quadratically with the number of streamlines, which can significantly affect the computational cost and memory requirements of generating and utilising this matrix. The relationship between streamline count and the multiple-comparison problem reflects a broader challenge shared also by voxel- and fixel-based analyses, where the dimensionality of the problem is dictated by the number of elements in the analysis mask. In typical whole-brain WM studies, voxel and fixel based methods typically involve ~400k-600k individual units at standard resolutions; while dimensionality can be lowered by reducing spatial resolution, doing so results in coarser anatomical detail in inferential outcomes. In SBA, the selection of ~350k streamlines for whole-brain coverage here yields a functional compromise between numbers of proximal streamlines for SSE (mean 740 streamlines using voxel grid 3mm and similarity threshold 0.1—see Methods) and redundancy in image sampling (mean 18 streamlines per fixel).

Although a one-to-one comparison with other methods is not possible given the fundamentally different domains in which analyses are performed (e.g. fixels vs streamlines) and quantitative metrics employed (e.g. FDC vs sFDC), the parallel application of SBA and FBA to the same cohort provides a useful reference. In the context of healthy ageing, FBA and SBA revealed broadly similar patterns of reduced WM connectivity consistent with known age-related WM vulnerability. Where the methods differed primarily in this respect is in the spatial coherence and anatomical interpretability of these results. With FBA, significant fixels can be fragmented and highlight only subset of a bundles (particularly in regions with complex fibre architecture or at tissue boundaries^37^), and association between significant effects and WM anatomy is somewhat subjective based on the locations and fibre orientations of fixel clusters and their assignment to specific WM bundles. In contrast, SBA intrinsically yields significance attributable to entire WM pathways as defined by streamlines trajectories and endpoint locations, which is beneficial for both visualisation and objective anatomical labelling. Importantly, this emphasis on streamline trajectories makes SBA particularly well-suited to a broad range of neuroscience and clinical research questions in which effects are manifested along WM pathways. By operating agnostically to the specific streamline-wise quantitative metric, SBA can capture distributed alterations across diverse conditions. For example, it is well-suited to studying processes such as Wallerian degeneration propagating along affected axons in Multiple Sclerosis^38^, more diffuse patterns of microstructural degradation seen in Cerebral Small Vessel Disease^39^, as well as neurodevelopmental trajectories in typical^40^ versus atypical^41^ populations. More generally, the SBA framework provides a natural way to investigate any scenario where changes manifest coherently along white matter pathways, including common neurological, psychiatric, and developmental conditions, thereby broadening its relevance beyond niche applications.

Despite the discussed benefits, SBA entails possible limitations that merit consideration. The SBA outcome is inherently affected by, and sensitive to, the limitations of the mechanism employed to construct the streamline-wise metric of interest, varying based on factors such as the choice of the imaging metric being sampled (which could be voxel- or fixel-based), and computation of a per-streamline aggregate of such. For example, a biological effect localised to one bundle may result in significant streamline-wise reports for intersecting pathways that sample from the same image locations. Consequently, a significant streamline does not guarantee an effect along its entire trajectory, and therefore should not be interpreted as such. Notably, a fixel-specific approach only partially alleviates this issue compared to voxel-based metrics given the possible convergence of distinct bundles within a fixel, also known as the “bottleneck” effect^37^. Moreover, while the sensitivity to image registration is a shared vulnerability across statistical inference frameworks (VBA, FBA, SBA), the implications in SBA are distinct: misalignment between template and subject spaces can lead to sampling from inappropriate vertex locations. The proposed back-tracking/re-tracking refinement alleviates this, but rigorous data quality control remains pertinent. Furthermore, because the framework utilises a template tractogram, its sensitivity is bounded by its representation of WM architecture; biological effects can only be detected in pathways adequately reconstructed within the template tractogram. State-of-the-art tracking methods are therefore recommended^42^.

Within the broader landscape of white matter analysis methods, SBA offers a distinct and complementary perspective by operating directly at the level of individual streamlines. In addition to previously mentioned methods (i.e. voxel-, fixel-, tract-level), SBA can also complement tractometry approaches, which characterise microstructural variation along predefined tract profiles^43,44^, by extending analyses beyond predefined bundles. Similarly, while structural connectomics captures the topology of large-scale brain networks^45,46^, SBA provides a means to link such network-level organisation to underlying imaging-derived, streamline-wise properties.

In conclusion, these observations establish SBA as an anatomically grounded, statistically rigorous framework for whole-brain, streamline-wise statistical inference. By unifying pathway-level specificity, high-resolution localisation, and FWE–controlled inference, SBA fills an important analytical gap between voxel-based, fixel-based, and whole-tract approaches. Its flexibility, compatibility with diverse imaging metrics, and natural alignment with the neuroanatomical structure of WM, suggest that SBA can serve as a powerful complementary tool to investigate pathway-wide alterations, while retaining streamline resolution, and thus provide a new avenue to address diverse research and clinical questions.

## Methods

### Data set

Data from the public Human Connectome Project in Aging (HCP-A)^29^ were used in this study to illustrate the performance of the SBA method *in vivo*. Subjects from two age groups were selected to investigate age-related changes. In accordance with the HCP-A classification, the subjects were grouped as “mature” (age 37.36 ± 0.65) and “old” (age 74.67 ± 0.94), with each group consisting of 30 subjects randomly selected with the constraint of being evenly split by sex (total sample size n=60). Structural data comprised both T1-weighted (T1w) and T2-weighted (T2w) images acquired at 0.8 mm isotropic spatial resolution. The T1w scans used a multi-echo MPRAGE sequence^48^ (TEs=1.8/3.6/5.4/7.2 ms, TI=1.0 s, TR=2.5 s). A variable-flip-angle turbo spin echo acquisition protocol^49^ was used for the T2w scans (TE=564 ms, TR=3.2 s). The dMRI acquisition protocol used 199 volumes across 3 b-values (14/92/93 directions for b=0/1500/3000 s/mm^2^, respectively) with a 1.5 mm isotropic resolution. The dMRI protocol also included a complete set of volumes acquired with reverse phase-encoding polarity for correcting susceptibility-related image distortions^50^. Further details about the HCP-A imaging protocol were previously described^29^.

### Preprocessing

While structural data were used in their HCP minimally preprocessed form^51^, dMRI data were instead obtained in raw form and subjected to a custom preprocessing pipeline tailored to the requirements of the subsequent analysis. Specifically, after initial denoising^52–54^ and removal of Gibbs ringing artifacts^55,56^, data were corrected for eddy current-induced distortion and subject movement^57^, as well as susceptibility-induced field distortion^50,58^. B1 field inhomogeneity correction^59^ and whole-brain masking of the dMRI data^60^ were applied. Then, dMRI data were upsampled to the structural data resolution using a B-spline interpolation to increase anatomical contrast^61^. The listed preprocessing steps were performed using MRtrix implementations or wrapper scripts^62^. Finally, image registration between structural and dMRI data was conducted to ensure anatomical alignment^63^.

### Template construction

A tissue-unbiased multi-modal approach to constructing a study-specific template tractogram was adopted to ensure good alignment of core WM bundles, peripheral WM structures, and cortical and subcortical GM regions^19^. Template construction at 0.8 mm isotropic spatial resolution was performed using T1w, T2w, Fractional Anisotropy (FA), and Mean Diffusivity (MD)^64^ data from the entire study cohort. The FA and MD scalar maps were derived by fitting a diffusion tensor model to the pre-processed dMRI images^65^. Fibre Orientation Distributions (FODs) were estimated for each subject using the multi-shell multi-tissue constrained spherical deconvolution technique^66,67^ and a population-averaged response function^60,68^; these were then warped to the template space and averaged to obtain a template FOD image. A template tractogram of one million streamlines was generated from this image utilising the iFOD2 algorithm^69^ and dynamic seeding^70^; Anatomically-Constrained Tractography^71^ was additionally imposed utilising a tissue segmentations based on the T1w template image. The tracking parameters were as currently suggested for the MRtrix3 FBA pipeline (https://mrtrix.readthedocs.io/en/3.0.8/fixel_based_analysis/mt_fibre_density_cross-section.html) (streamline length 10–250mm; maximum turning angle 22.5 degrees; FOD power 1.0; all others default).

The SIFT method was applied until convergence^20^. In addition to reducing the total streamline count to a computationally feasible number, the fact that this method improves the biological accuracy of the tractogram reconstruction by matching streamline densities to the underlying apparent fibre density (here as computed from the template FODs) results in improved homogeneity of streamline coverage across the breadth of possible trajectories, reducing streamline redundancy and stabilising the numbers of proximal streamlines across the tractogram.

### Subject-specific tractogram refinement

Further refinement steps were taken to explicitly conform streamlines to subject-specific anatomy, while still retaining streamline correspondence between subjects. First, a tissue segmentation was derived^73^ for each subject, which provided the anatomical classification used to exclude template tractogram streamlines that did not intersect WM (typically short and U-shaped streamlines that ended up fully within cortex in subject space). This step ensured that all retained template streamlines traversed WM across the entire study cohort. Second, where possible we performed a regeneration of the terminal portions of the template streamlines in subject space (see “Back-tracking/Re-tracking Algorithm” in Supplementary Information); this is conceptually similar to the previously described ‘back-tracking’ method^71^. The back-tracking/re-tracking approach allows each projected template streamline to adjust the trajectory of its distal portion to better match the subject-specific FOD field and tissue segmentation, resulting in superior sampling of subject-specific image data underlying those trajectories while preserving streamline correspondence across subjects. To quantitatively assess the performance of the back-tracking/re-tracking algorithm, we compared both the template tractogram projected to subject space and its refined version with a “native” tractogram generated in subject space, which is only used here as benchmark for evaluating the template tractogram versions match with the morphology in subject space (note however the native tractogram plays no role in the SBA pipeline). For each tractogram, streamline density maps were generated at 0.5 mm isotropic resolution by summing the SIFT2 multipliers^70^ within each voxel^23^. The Mean Square Error was then calculated to quantify the divergence from the native tractogram TDI benchmark.

### Streamline-wise quantification and statistical modelling

In the SBA framework, the tractogram serves as the domain for statistical analysis, with template streamlines acting as the fundamental units for metric quantification. To derive quantitative measures for each streamline, we employed a strategy consistent with the Track-Weighted Imaging method^24^, where a metric of interest is sampled at each vertex of the streamline, and a per-streamline summary statistic is computed. In this study, for illustration, we focused on the combination of microstructural and macrostructural features commonly used in FBA. We first computed along-streamline averaged Fibre Density^68^ (sFD) and Fibre Cross section^25^ (sFC), and then defined the streamline fibre density and cross section (sFDC) as follows:

(1) sFDC=sFD⋅sFC

Group-level differences in sFDC between the mature and old cohorts were estimated using a General Linear Model (GLM)^74^. Sex and brain volume were selected as nuisance covariates to control any potential confounding influence arising from those features^75–77^. Skull stripping and CSF exclusion^73,78^ were performed before computing the brain volume to account for structural brain changes that occur with healthy ageing, e.g. ventricular enlargement and sulcal widening^34^. The resulting volumes were demeaned and normalized to unit variance.

### Streamline similarity

Establishing a similarity metric between streamlines is a requisite step in the SBA framework to enable both data smoothing and statistical enhancement. The implemented similarity criterion is based on the overlap of their voxel visitations, similar to that used previously in other contexts^26,28^. Specifically, a 3D Euclidean space was established as a 3mm isotropic voxel grid, and each grid element assigned a unique label to enable efficient mapping of streamline trajectories. The streamline spatial encoding w was then defined as the number of streamline vertices within each grid element, with unvisited voxels immediately adjacent to any visited voxel additionally allocated a value of 0.5 to provide mild unaliasing, normalised to a unit sum per streamline. To formalise this, we define the “primary” label set as the set of voxels containing at least one streamline vertex, and the “secondary” one as the set of immediately adjacent unvisited voxels (Extended Data Fig. 1). The pairwise similarity between streamline i and j follows:

(2) $$\varphi (i,j)=J(i,j)\cdot \left\langle w_{i},w_{j}\right\rangle$$

where J(i,j) represents the Jaccard index of the label sets $L_{i}$ and $L_{j}$ (the union of the primary and secondary label sets for streamlines i and j, respectively):

(3) $$J(i,j)=\frac{\left|L_{i}\cap L_{j}\right|}{\left|L_{i}\cup L_{j}\right|}$$

This composite formulation was derived to capture streamline similarity at both global and local scales. The Jaccard index functions as a set-theoretic scaling factor that accounts for the global spatial footprint and penalises partial overlaps. Conversely, the inner product term captures the correlation of visitation densities. By modulating the local visitation correlation with the global Jaccard index, the metric ensures that high similarity requires both global topological agreement and local trajectory coincidence. The similarity value is normalised to satisfy the condition that the self-similarity of any element is 1, enabling consistent interpretation across pairs, and resulting in a directed (non-symmetric) similarity measure (i.e. φ(i,j)≠φ(j,i)). Finally, pairwise similarities below 0.1 were considered negligible and omitted.

### Similarity-informed smoothing

Although the implementation of spatial smoothing is methodologically well-established through the use of a Gaussian kernel in voxel space^11^, between-streamline smoothing of streamline-wise data (e.g. sFDC) required adapting these principles to a different metric of spatial proximity. A spatial Gaussian kernel assigns higher weights to nearby voxels and lower ones to those farther away. In the context of streamline data, we use the pairwise similarity metric to drive this weighting, thereby achieving smoothing of the data associated to the streamline i as follows:

(4) $$s_{i_{\text{smoothed}}}=\frac{\sum_{j=0}^{N}s_{j}\cdot \omega (i,j)}{\sum_{j=0}^{N}\omega (i,j)}$$

(5) $$\omega (i,j)=e^{F_{j}}\cdot \varphi (i,j{)}^{D}$$

where N is the number of streamlines in the template tractogram. Therefore, the presented approach implements a local smoothing where the weight is defined as the pairwise streamline similarity metric φ from Equation (2) that is modulated by the power coefficient D and scaled by the $e^{F_{j}}$ multiplier from the SIFT2 method^70^. The per-streamline SIFT2 multipliers were optimised to match the template tractogram to the template fixel-wise fibre density. D was set to 5, which applies only mild smoothing (see Simulation Results in Supplementary Information concerning the choice of the D value).

### Similarity-informed streamline enhancement

We introduce an adaptation of the TFCE approach^16^ to perform streamline-wise test-statistic enhancement, a method we refer to as Similarity-informed Streamline Enhancement (SSE).

(6) $$\text{SSE}(j)=\frac{\int_{0}^{t_{j}}e(j,t{)}^{E}t^{H}dt}{e(j,0)}$$

(7) $$e(j,t)=\sum_{i=0}^{N}\theta \left(t_{i}-t\right)\cdot e^{F_{i}}\cdot \varphi (i,j{)}^{M}$$

where θ is the Heaviside function (1 if $t_{i}>t,$ 0 otherwise). As t progresses from 0 to the test-statistic $t_{j}$ of the streamline j, any streamline i with both a test statistic greater than t and a non-zero similarity to streamline iφ(i,j), contributes to the “cluster extent” e(j,t) supporting streamline j at cluster-forming threshold t. Contribution to the enhanced test statistic is determined by the product of e(j,t) and t, exponentiated by the statistical enhancement parameters E and H respectively (with H>1 placing greater emphasis on supra-threshold cluster formation at higher test statistic thresholds). These contributions are summed across a discrete range of cluster-forming thresholds t+=dt (so-called “threshold-free”), with dt=0.1. Normalization is performed to intrinsically address the effect of non-stationarity^79^, i.e. the difference in prevalence of similar streamlines, by adapting a correction approach previously introduced for FBA^47^. Recommended settings for E,H, and M are discussed in the “Simulation Results” section in Supplementary Information. The SIFT2 multipliers $e^{F_{i}}$ are the same employed in Equation (5).

### Statistical inference

Streamline-wise inference of statistical effect presents the challenge of maintaining adequate statistical power despite the high dimensionality of the analysis. To address this, we integrated SSE within a non-parametric permutation testing framework^17^, which permutes model residuals to rigorously account for nuisance variables^74^. Family-Wise Error (FWE) control was adopted as the method for multiple comparison correction to ensure a strict control over the probability of any false positives across the large number of tests performed. FWE-corrected p-values were obtained from the null distribution constructed from the maximum enhanced test statistic observed within each of the 5000 permutations performed (including the default permutation), as previously described^80^. The statistical inference employed a GLM^74^ to estimate group-level effects across the tractogram: a one-sided t-test was performed to assess whether the sFDC was significantly higher in the mature group compared to the old group with sex and brain volume included as nuisance variables (see “Streamline-wise Quantification and Statistical Modelling” for details). The reverse contrast (old > mature) was also tested but only yielded negligible non-meaningful effects, and therefore not reported further. While the SBA framework was here employed for testing group differences, through generality of the GLM it is capable of investigating associations with continuous or categorical predictors^74^.

### Fixel-based analysis

For comparison and evaluation of the information provided by the novel SBA method, the same data were also analysed using Fixel-Based Analysis (FBA)^13^, a well-established and widely used framework for detecting changes in WM metrics, both in the presence and absence of clinical conditions^4,25,81–84^. FBA enables quantification of WM metrics, data smoothing, and statistical inference at the so-called *fixel* level, where the term fixel refers to a fibre population within a voxel^2^ (i.e. a voxel can contain multiple fixels). For consistency, the FDC^25^ metric was used to test the same directional hypothesis (mature > old) as investigated with SBA, and using the same nuisance covariates as described previously (i.e. total brain volume and sex). FBA was performed using the MRtrix3 implementation^62^ and followed the recommended pipeline (https://mrtrix.readthedocs.io/en/3.0.8/fixel_based_analysis/mt_fibre_density_cross-section.html) utilising the same study-specific tissue-unbiased template previously described.

### Post-hoc analysis

We carried out a post-hoc analysis to further characterise the observed sFDC (for SBA) and FDC (for FBA) effects by disentangling the relative contribution of microstructural (FD) and macroscopic morphological (FC) changes. For SBA sFDC is computed as the product of along-streamline averages of FD and FC, here referred to as sFD and sFC respectively.

We computed per-fixel $\left(\alpha_{FBA}\right)$ and per-streamline $\left(\alpha_{SBA}\right)$ indices to quantify the relative influence of FD versus FC in both FBA and SBA contexts, similarly to previously described^30^:

(8) $$\alpha_{FBA}=\frac{\Delta_{FD}\cdot \mu_{FDC}}{\Delta_{FDC}\cdot \mu_{FD}}$$

(9) $$\alpha_{SBA}=\frac{\Delta_{sFD}\cdot \mu_{\text{sFDC}}}{\Delta_{\text{sFDC}}\cdot \mu_{sFD}}$$

where Δ denotes the GLM-predicted difference in means of the mature and old groups, and μ the predicted mean of the mature group, both accounting for nuisance variables. While these measures are unbounded, we constrained them to the [0, 1] interval to facilitate interpretation and visualisation. Values of α<0.5 indicate that observed sFDC (or FDC) changes are predominantly driven by macrostructural (sFC or FC) differences, whereas α>0.5 indicate a stronger microstructural (sFD or FD) contribution. Extremes (α=0 or α=1) correspond to entirely FC- or FD-driven effects respectively.

### Simulations for parameter optimisation

To provide an informed recommendation of suitable parameters to use in SBA applications, we used a Receiver Operator Characteristic (ROC)-based evaluation^85^ on synthetic test-statistic data to compare the performance of the SBA smoothing and enhancement across different configurations.

The overall simulation pipeline (see Supplementary Information for further details) is an adaptation of those previously performed to assess TFCE^16^ and Connectivity-based Fixel Enhancement (CFE)^2^. In brief, the similarity-informed smoothing power coefficient D from Equation (5) was tested for a range of values (D∈[0,0.5,1,2,5,∞]), where infinity denotes the omission of the smoothing step. For the SSE process, we varied E∈[0.5,1,1.5,2,3,4,5,6] and H∈[0.5,1,2,3,4,5,6] from Equation (6), as well as M∈[0,0.25,0.5,0.75,1,1.5] from Equation (7). We ran the pipeline on four different simulated effect patterns and at three different Signal-to-Noise Ratio (SNR) values (SNR=[1,2,3]). Additionally, we considered two streamline-wise metrics, which are expected to exhibit different intrinsic covariance structures: along-streamline averaging, previously described; endpoint averaging, in which only the samples at the streamline terminal points were used. All combinations of these configurations were evaluated, resulting in a total of 48,384 tests used to determine recommended parameter values for SBA applications (E=2,H=3,M=0.25,andD=5). Synthetic data generation, simulation pipeline, and simulation results are reported in full in the Supplementary Information.

## Extended Data

**Extended Data Fig. 1.**
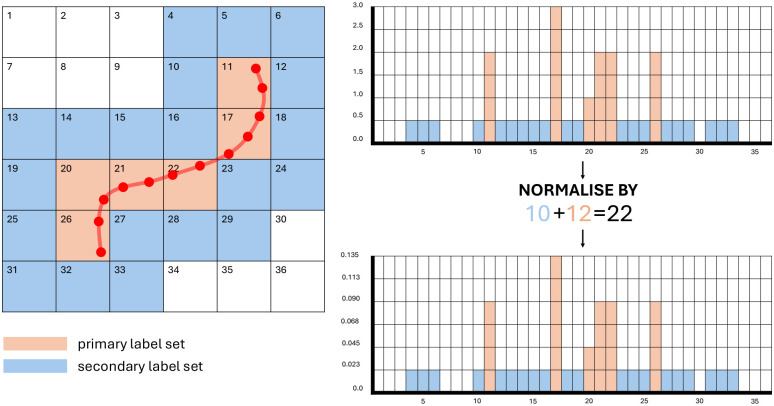
Example of the encoding of a streamline pathway for pairwise streamline similarity computation. **Left**: Streamline vertices are assigned labels corresponding to an underlying label grid, where orange denotes the primary label set (weight=1), whereas blue regards the secondary one (weight=0.5). **Top right:** A histogram of absolute frequencies is constructed from these labels. **Bottom right:** The sum of bin counts is computed and used to normalize the absolute frequencies, yielding relative frequencies.

## Supplementary Material

Supplementary Files

This is a list of supplementary files associated with this preprint. Click to download.

- SupplementaryVideo1.mp4
- StreamlineBasedAnalysisSupplementaryInformation.pdf
- SupplementaryVideo2.mp4

## Data Availability

The data used in this study were obtained from the Human Connectome Project in Aging database (https://www.humanconnectome.org/study/hcp-lifespan-aging), and a list of the unique subject identifier codes relevant to this study is provided in a Zenodo repository (https://doi.org/10.5281/zenodo.20654825).

## Code availability

The relevant software used to perform the presented analysis is available through the Zenodo open-access repository (https://doi.org/10.5281/zenodo.20654825). Ongoing updates and community contributions are also tracked via a pull request on the MRtrix3 GitHub (https://github.com/MRtrix3/mrtrix3/pull/3303), which was active at the time of submission. Finally, a separate repository (https://github.com/simzanoni/SBA) provides a step-by-step tutorial for users wishing to apply Streamline-Based Analysis to their own data.
